# Lid Films of Poly(3-hydroxybutyrate-*co*-3-hydroxyvalerate)/Microfibrillated Cellulose Composites for Fatty Food Preservation

**DOI:** 10.3390/foods12020375

**Published:** 2023-01-13

**Authors:** Eva Hernández-García, Amparo Chiralt, Maria Vargas, Sergio Torres-Giner

**Affiliations:** Research Institute of Food Engineering for Development (IIAD), Universitat Politècnica de València (UPV), 46022 Valencia, Spain

**Keywords:** PHBV, cellulose, sustainable packaging, food quality and shelf life, migration

## Abstract

The present work evaluates the food packaging performance of previously developed films of poly(3-hydroxybutyrate-*co*-3-hydroxyvalerate) (PHBV) reinforced with atomized microfibrillated cellulose (MFC) compatibilized by a reactive melt-mixing process. To this end, the novel green composite films were originally applied herein as lids in aluminum trays to preserve two dissimilar types of fatty foods, namely minced pork meat and sunflower oil. Results indicated that the PHBV/MFC films effectively preserved the physicochemical and microbiological quality of pork meat for one week of storage at 5 °C. In particular, the compatibilized green composite lid film yielded the lowest weight loss and highest oxidative stability, showing values of 0.935% and 0.78 malonaldehyde (MDA)/kg. Moreover, none of the packaged meat samples exceeded the acceptable Total Aerobial Count (TAC) level of 5 logs colony-forming units (CFU)/g due to the improved barrier properties of the lids. Furthermore, the green composite films successfully prevented sunflower oil oxidation in accelerated oxidative storage conditions for 21 days. Similarly, the compatibilized PHBV/MFC lid film led to the lowest peroxide value (PV) and conjugated diene and triene contents, with respective values of 19.5 meq O_2_/kg and 2.50 and 1.44 g/100 mL. Finally, the migration of the newly developed PHBV-based films was assessed using two food simulants, proving to be safe since their overall migration levels were in the 1–3 mg/dm^2^ range and, thus, below the maximum level established by legislation.

## 1. Introduction

Food preservation aims to extend shelf life and provide safer products to consumers using varied materials and technologies [1]. In this sense, packaging solutions based on plastics have been extensively used due to their transparency, flexibility, ease of processing, reduced cost, low weight, and high versatility [2]. In addition, packaging materials can provide other functions to the food product, such as active and bioactive properties [3]. Nowadays, the development of sustainable materials and designs represents a fundamental challenge in the food packaging industry [4,5]. However, most of the conventional materials currently used for food packaging are based on non-biodegradable petrochemical polymers that cause high environmental impacts related to their disposal.

A possible packaging alternative approach is the use of biopolymers, which are macromolecules that are either obtained from natural sources or biodegradable or show both features [6,7,8,9,10]. Packaging materials based on biopolymers can be feasibly composted in industrial facilities and, in some cases, in domestic composts and natural environments [11,12]. Among biopolymers, polyhydroxyalkanoates (PHAs), such as poly(3-hydroxybutyrate) (PHB), are semi-crystalline aliphatic polyesters obtained from microorganisms that rapidly biodegrade in composting facilities and even in the environment [13]. PHB is a homopolyester that shows physical properties similar to those of polypropylene (PP) and polyethylene terephthalate (PET) [14]. PHB shows high crystallinity and low thermal stability, yielding rigid and brittle materials, which limits its use in food packaging applications [15]. However, the copolymer of 3-hydroxybutyrate (3HB) with 3-hydroxyvalerate (3HV), that is, poly(3-hydroxybutyrate-*co*-3-hydroxyvalerate) (PHBV), shows higher ductility and reduced crystallinity, opening up new possibilities in food packaging [16].

Furthermore, a newly designed strategy for improving the performance of PHBV-based packaging materials within the framework of the Circular Economy is its reinforcement with renewable and biodegradable micro- or nanostructured fillers, such as microfibrillated cellulose (MFC) [17]. In this sense, an innovative and scalable technique was developed in a previous study of the research group to prepare green composite films based on PHBV and atomized MFC [18]. Atomization, also called the spraying method, is a process in which emulsions or other multiphase systems are broken into small drops of liquid by high-speed fluids (typically compressed gas) or fluids with centrifugal force, and then solidified into powder. The atomization process of MFC suspensions resulted in the formation of ultrathin cellulose structures, having a rod-like morphology and sizing approximately 3 ± 1 µm, which were composed of fibers with thicknesses in the nano-range. The atomized MFC particles were thereafter melt-mixed with PHBV and subsequently processed into films by thermo-compression. Incorporating the nanostructured fibers of cellulose successfully improved the physical properties of the biopolyester, mainly in terms of tensile and gas barrier properties, without reducing thermal stability or significantly altering the optical properties. Moreover, these green composite formulations were compatibilized with different reactive additives to enhance the filler-to-matrix interfacial adhesion and, hence, their performance for food packaging applications. Among all the range of formulations tested, optimal results were attained for PHBV containing intermediate MFC content of 5 wt% and compatibilized with the combination of triglycidyl isocyanurate (TGIC) with dicumyl peroxide (DCP) by a reactive melt-mixing process.

Thus, the present study focuses on applying the previously prepared compatibilized green composite films as rigid lids for food packaging applications. To this end, two types of fatty foods were selected: minced pork meat and sunflower oil. On the one hand, pork meat is a high-protein food of high biological value. It also contains varying amounts of lipids, including saturated fatty acids, which usually range from 2.5 to 11.9 g/100 g of meat and can be much higher depending on the level of trimming and other factors [19]. Minced pork meat is known to deteriorate during storage due to microbial spoilage, surface dehydration, and myoglobin and lipid oxidation, which could decrease its nutritional value and cause changes in appearance and production of off-flavors and odors [20,21]. On the other hand, sunflower oil is rich in unsaturated fatty acids, mainly oleic and linoleic [22]. These are associated with oxidation that can modify the organoleptic properties of the oil and cause losses in nutritional value and quality [23]. Therefore, in both foods, lipid oxidation should be prevented or at least delayed [24], and the PHBV/MFC composites can be regarded as excellent candidates to accomplish this objective due to their improved barrier against oxygen. Therefore, green composites of PHBV with 5 wt% MFC, with and without compatibilizers, were prepared in the form of films and applied as lids in trays containing the fatty foods. The quality and shelf-life of the two packaged foodstuffs were evaluated throughout storage and compared with films of neat PHBV and a commercial high-barrier multilayer habitually used in the food packaging industry. Finally, migration studies were performed using food simulants to corroborate the safety and potential use of green composite films in food packaging.

## 2. Materials and Methods

### 2.1. Materials

Commercial PHBV with food-grade status was supplied as ENMAT Y1000P in the form of pellets by Tianan Biologic Materials (Ningbo, China). The 3HV fraction in the copolyester is ~2 mol%, and the molecular weight (M_W_) is ~2.8 × 10^5^ g/mol. The aqueous suspension of nanocellulose, Exilva F01-V grade, was provided by Borregaard ChemCell (Barcelona, Spain) in the form of a gel with a solid content of 10 wt%. The compatibilizers TGIC, reference 379506, with molecular weight (M_W_) of 297.26 g/mol, and DCP, reference 329541, with M_W_ of 270.37 g/mol and 98% purity, were purchased from Sigma-Aldrich S.A. (Madrid, Spain). Commercial high-barrier multilayer film, based on polyamide 6 (PA6) and poly(ethylene-*co*-vinyl alcohol) (EVOH), with a total thickness of 140 µm, was supplied by WK THOMAS (Barcelona, Spain).

Minced pork meat (Consum S. Coop. V., Valencia, Spain) and sunflower oil (maximum acidity 0.2°, average values per 100 mL: energy value: 3397 kJ/826 kcal; fats: 92 g (7.3 g saturated, 50 g monounsaturated, and 34.3 g polyunsaturated); 0 g carbohydrates; 0 g proteins; 0 g salt) (Mercadona, Valencia) were purchased from local supermarkets. Trichloroacetic acid (TCA) (99.5% purity), 2-thio-barbituric acid (TBA) (>98% purity) reagent, glacial acetic acid (99.7% purity), sodium thiosulfate (Na_2_S_2_O_3_) (99.5% purity), and isooctane (99.5% purity) were supplied by Panreac Química, SA (Castellar del Vallés, Spain). Iodine (99.5% purity) and 1-decanol (≥99.0% purity) were obtained by Acros Organics^®^ (Geel, Belgium) and Sigma-Aldrich (Madrid, Spain), respectively. Potassium iodide (≥99.0% purity) were supplied by Sigma-Aldrich (Madrid, Spain). Microbiological media: buffered peptone water, Violet Bile Red agar (VRB), and Plate Count agar (PCA) were provided by Scharlab S.A. (Barcelona, Spain), whereas Man, Rogosa, and Sharpe agar (MRS) were supplied by Lankem-Labbox (Barcelona, Spain).

### 2.2. Preparation of PHBV-Based Green Composites

The green composite films were prepared using PHBV and the MFC powder, which was obtained by atomization in a laboratory with spray-drying equipment (Minispray Dryer Büchi B-290 Advanced, BUCHI Ibérica S.L.U., Barcelona, Spain) using compressed air in previously optimized conditions [18]. The PHBV pellets were first dried in a vacuum oven (vacuum TEM-TJP Selecta, S.A., Barcelona, Spain) at 60 °C and 0.2 bar for four hours. Afterward, the dried pellets and MFC powder were placed in a dissector containing phosphorus pentoxide (P_2_O_5_, 99% purity, reference 214701, Sigma-Aldrich, S.A.) at 25 °C for one week to remove the remaining water. Then the PHBV pellets were melt-mixed with MFC at 5 wt% in an internal mixer (HAAKE™ PolyLab™ QC, Thermo Fisher Scientific, Herzogenaurach, Germany) at 170 °C and 50 rpm. The mixing time was set at 5 min after analyzing the stability of neat PHBV during melt-mixing. The processed amount of each composition was approximately 50 g. PHBV samples with MFC and compatibilizers (TGIC and DCP) were used to produce the green composites (PHBV/MFC + TGIC + DCP). Samples without MFC with compatibilizers (PHBV + TGIC + DCP), with MFC without compatibilizers (PHBV/MFC), and neat PHVB were also prepared as control materials. Table 1 summarized the set of formulations prepared. 

The samples obtained from the internal mixer were cold-milled to have the product in powder, an easier to handle format for subsequent pressing. To this end, each dough was ground at two pulses of 30 s in a milling machine (Model M20, IKA, Staufen, Germany). The powder-like material from the milling process was stored in a desiccator at 25 °C and 0% relative humidity (RH) with silica gel for one week. It was necessary to use 2.5 g of PHBV powder to obtain a film sizing 10 cm × 10 cm with a thickness of approximately 130 µm. Compression molding was carried out in a hydraulic press (Model LP20, Labtech Engineering, Bangpoo, Thailand) by placing the samples between two sheets and inside a square frame of Teflon. The samples were preheated for 3 min at 200 °C without pressure, then for 4 min pressed at 200 °C and 100 bar, and finally cooled for 3 min until 80 °C. The resultant films were cut into round disks of approximately 7 cm in diameter, separated by absorbent laboratory paper, and stored in a desiccator with P_2_O_5_ (0% RH) at 25 °C. All the film samples remained in the desiccator for a minimum of 15 days to eliminate the remaining humidity and reduce the effect of physical aging of PHBV before using them as packaging lids.

### 2.3. Evaluation of Packaged Foods

All devices and work surfaces were first disinfected with 96% ethanol (Panreac S.A, Barcelona, Spain). All the packaging films were sterilized by exposure to ultraviolet (UV) light for one hour in a laminar flow cabinet (Bio II Advance, Telstar, Terrasa, Spain). The films were used as lids in aluminum cups sizing 6.6 cm in diameter and 2.4 cm in height (WPAL-050-100, Quima S.L, Valencia, Spain) in order to evaluate their effect on the food shelf life. The films were sealed manually using an extra instant adhesive glue (Pattex Crocodile, Ferreteria Moreno, Valencia, Spain). All food samples were packaged and handled inside the laminar flow cabinet.

#### 2.3.1. Minced Pork Meat 

Minced pork meat samples (10 g) were immediately transported after purchase to the laboratory facilities in a portable fridge at 5 °C and placed in the aluminum cups that were closed with lids made of the various films. All samples were stored for up to 7 days at 5 °C and 48% RH, and the packaged pork meat samples were analyzed in terms of weight loss, pH, lipid oxidation, color changes, and microbial counts.

After 3 and 7 days of storage, the food samples were weighed to determine, in triplicate, their weight loss using an analytical balance (ME36S, ±0.0001 g accuracy, Sartorius, Goettingen, Germany). The pH values were also determined using a digital pH meter by directly inserting the electrode probe (Mettler-Toledo GmbH, Schwerzenbach, Switzerland) into the pork meat sample. Five measurements were taken for each packaging treatment and time. The oxidative stability of pork meat was monitored using the thiobarbituric acid reactive substances (TBARS) at the beginning and end of the storage, following the methodology described by Siu & Draper [25]. To this end, each packaged pork meat sample was taken from the trays and placed in bags (Stomacher 440 Classic Strainer Bags, Worthing, UK) with 50 mL of distilled water and homogenized for 2 min using a homogenizer (Masticator Paddle blender, IUL Instruments, Barcelona, Spain). Then, 50 mL of 10% *v*/*v* TCA was added, and the homogenate was filtered with a vacuum pump using Whatman #1 filter paper (Whatman Nº1, Whatman International Ltd., Kent, UK). After that, 8 mL of the clear filtrate was added to 2 mL of 0.06 M TBA reagent and incubated for 90 min at 80 °C. The absorbance was read at 532 nm, and the results were obtained in triplicate and expressed as mg of malonaldehyde (MDA) per kg of meat sample.

The CIE L*a*b* color coordinates of the packaged pork meat were obtained using the illuminant D65/10° observer from the reflection spectra of the sample surface using the MINOLTA colorimeter spectrum (model CM-5, Minolta Co., Tokyo, Japan). Measurements were performed by placing the colorimeter on six random points of the lid films that were used to preserve the meat samples in the trays. The lightness (L*) and the color coordinates a* and b* in the CIE L*a*b* color space were determined. The lightness value, L*, defines black at 0 and white at 100. The a* axis represents green–red color (negative values toward green and positive toward red), whereas the b* axis represents blue–yellow color (negative values toward blue and positive toward yellow). The chroma (C_ab_*) and hue (h_ab_*) were determined by using Equations (1) and (2), respectively. The total color difference at the different storage times with respect to the initial day was calculated using Equation (3). Three packaged pork meat samples with each packaging film were analyzed in duplicate after 3 and 7 days of storage.

(1) $$C_{ab}^{*}=\sqrt{{\left(a^{*}\right)}^{2}+{\left(b^{*}\right)}^{2}}$$

(2) $$h_{ab}^{*}=arctg\frac{b^{*}}{a^{*}}$$

(3) $$\Delta E=\sqrt{{\left(\Delta L^{*}\right)}^{2}+{\left(\Delta a^{*}\right)}^{2}+{\left(\Delta b^{*}\right)}^{2}}$$

Microbiological analyses of the packaged pork meat samples were carried out after the different storage times (0, 3, and 7 days). The packaged meat samples were aseptically taken from the container in the laminar flow cabinet and subsequently placed in sterile bags with 90 mL of peptone water (Scharlab S.A., Barcelona, Spain). The stomacher bags were homogenized for 3 min in the homogenizer, and the resultant homogenate was then 10-fold serially diluted using Tryptic Soy Broth (TSB) (Scharlab S.A., Barcelona, Spain) and used to enumerate total viable counts, total coliforms, and lactic acid bacteria. The total aerobic counts (TAC) and total coliforms were determined on PCA and VRB plates, respectively, after incubation at 37 °C for 48 h. The lactic acid bacteria (LAB) were enumerated using MRS plates after incubation at 30 °C for 72 h. After incubation, all the colonies were counted, and the results were expressed as colony-forming units per gram (CFU/g). All the microbial tests were performed in triplicate.

#### 2.3.2. Sunflower Oil

Sunflower oil (50 mL) was placed in the aluminum trays and closed with lids made of the different films. The trays were stored for up to 21 days at 30 °C and 53% RH and exposed to a fluorescent light (model T8-G13, Leroy Merlin, Valencia, Spain) at an intensity of 1000–1500 lux, measured using a digital Luxometer (model RS Pro ILM1332A, RS Components, Madrid, Spain). The oxidative stability of the sunflower oil was measured in terms of the peroxide value (PV) and diene and triene content after 0, 4, 11, and 21 days of storage. 

The titrimetric method was used to determine the PV of the samples using an automatic titrator (Titrando, Metrohm Ion Analysis, Switzerland) [26]. To this end, 1 g of sunflower oil was dissolved in 10 mL of solvent (glacial acetic acid:1-decanol volume ratio of 3:2, containing 10–15 mg/L of iodine) and mixed with 200 μL of an oversaturated potassium iodide solution (130 g potassium iodide dissolved in 100 mL of distilled water). The mixture was thoroughly sacked and kept in the dark for 1 min. Then, 50 mL of distilled water was added, and the solution was titrated with 0.01 M or 0.001 M Na_2_S_2_O_3_, depending on the PV predicted. A blank control sample, without sunflower oil, was also prepared following the same procedure. All the analyses were performed in triplicate.

The conjugated dienes and trienes were also determined utilizing a spectrophotometric method [27]. The absorbance of the appropriately diluted samples in isooctane was measured in a spectrophotometer (Evolution 201 VisibleUV, ThermoScientific, Herzogenaurach, Germany) at wavelengths of 232 nm and 268 nm to determine the conjugated dienes and trienes, respectively.

### 2.4. Migration Tests

The overall migration levels of the PHBV-based films were evaluated in two food simulants, 10% *v*/*v* ethanol (simulant A) and olive oil (simulant D2), by immersing 0.5 dm^2^ of film sample in 50 mL of simulant at the normalized conditions of 40 °C for ten days. Total migration in 10% ethanol was evaluated by monitoring sample weight loss, whereas in the case of the vegetable oil it was obtained by extraction of the oil absorbed in the film sample followed by quantification by Gas Chromatography with Flame-Ionization Detection (GC-FID, GC 2400, PerkinElmer España SL, Madrid, Spain), according to ISO 1186 [28] and [29] standards, respectively. Three replicates per sample in each simulant were tested and the migration results were averaged.

### 2.5. Statistical Analysis

The statistical analyses of the data were performed through an analysis of variance (ANOVA) using Statgraphics Centurion XVII-X64 software (Manugistics Corp., Rockville, MD, USA). One-way and multifactor ANOVA were used to analyze the influence of the kind of packaging and storage time on the properties of packaged food. Fisher’s least significant difference (LSD) procedure was used at the 95% confidence level.

## 3. Results and Discussion 

### 3.1. Physicochemical Properties of Packaged Minced Pork Meat

**Figure 1:**
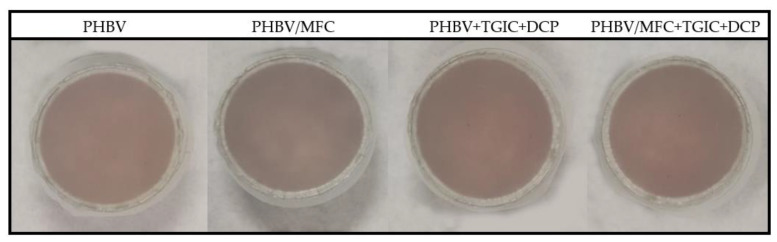
Images of the minced pork meat samples packaged in trays with the green composite lid films of poly(3-hydroxybutyrate-*co*-3-hydroxyvalerate) (PHBV) with microfibrillated cellulose (MFC) and compatibilized with triglycidyl isocyanurate (TGIC) and dicumyl peroxide (DCP).

Figure 1 shows the visual aspect of the packaged minced pork meat according to the type of lid film based on PHBV. All the samples showed a similar appearance, presenting low transparency due to the inherently high crystallinity of the microbial copolyester. 

**Figure 2:**
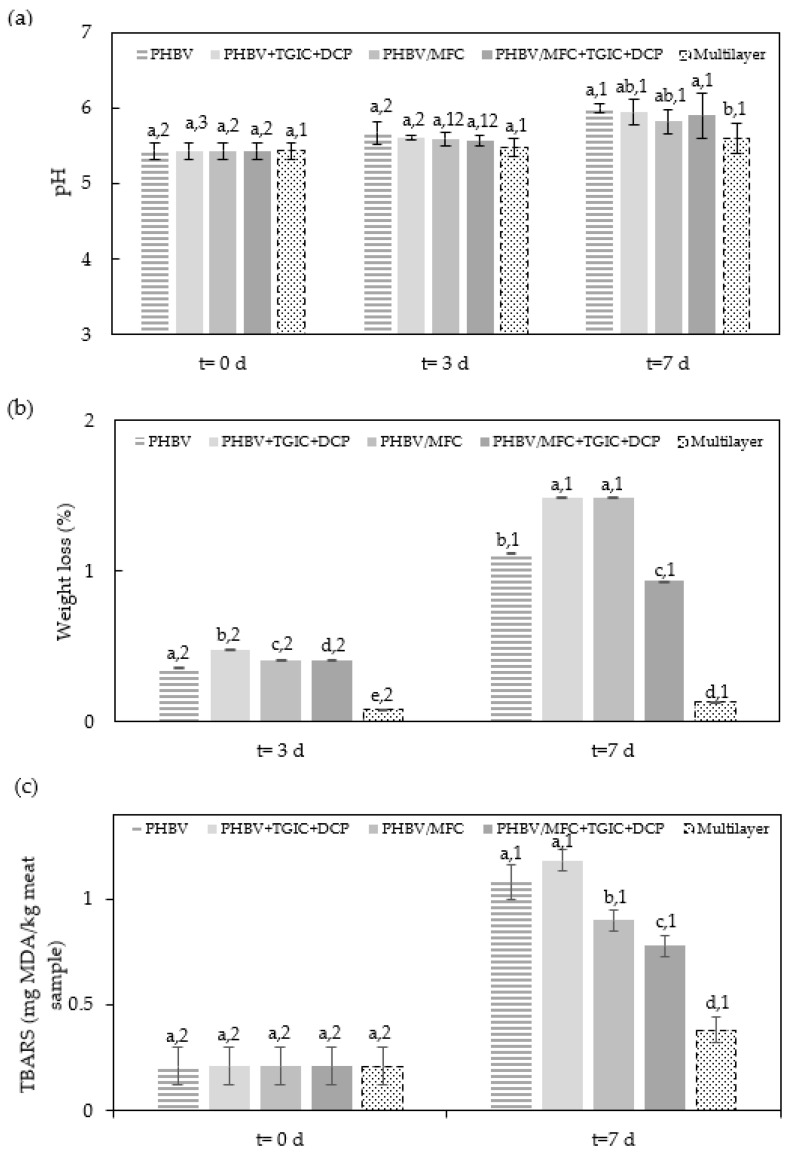
(**a**) Changes in pH, (**b**) weight loss, and (**c**) thiobarbituric acid reactive substances (TBARS) of the cold-stored minced pork meat samples packaged in trays with the green composite lid films of poly(3-hydroxybutyrate-*co*-3-hydroxyvalerate) (PHBV) with microfibrillated cellulose (MFC) and compatibilized with triglycidyl isocyanurate (TGIC) and dicumyl peroxide (DCP) and the multilayer commercial film. Oxygen permeability (OP, m^3^·m·m^−2^·Pa^−1^·s^−1^) and water vapor permeability (WVP, kg·m·m^−2^·Pa^−1^·s^−1^) data of each film reported in previous studies * [18] and ** [21]: PHBV (OP* = 2.09 × 10^−19^ and WVP* = 1.84 × 10^−15^), PHBV + TGIC + DCP (OP* = 2.70 × 10^−19^ and WVP* = 5.69 × 10^−15^), PHBV/MFC (OP* = 1.24 × 10^−19^ and WVP* = 3.29 × 10^−15^), PHBV/MFC + TGIC + DCP (OP* = 0.98 × 10^−19^ and WVP* = 3.29 × 10^−15^), and Multilayer (OP** = 2.74 × 10^−20^ and WVP** = 7.95 × 10^−16^, assuming monolayer). Various superscripts (letters) indicate significant differences among samples for the same storage time (a–e) and superscript numbers due to storage time for the same sample (1–3) (*p* < 0.05).

In order to quantify the physicochemical properties of the packaged minced pork meat during storage, Figure 2 shows the changes in pH, weight loss, and TBARS according to the type of lid film. The figure note also includes the corresponding values of water vapor and oxygen permeability, since the physicochemical properties of the packaged foods are related to the barrier performance of the films. It can be seen in Figure 2a that the initial pH of the pork meat samples was 5.43 ± 0.11, which is in the range of the values previously reported for fresh pork meat samples [30]. As expected, the pH values of meat increased during storage due to the increased content of nitrogenous bases resulting from proteolysis caused by the activity of microorganisms [31]. This increase, slightly lower in the meat samples packaged in trays with the commercial high-barrier lid film, the so-called multilayer sample, was not affected significantly (*p* > 0.05) by any of the evaluated PHBV samples. The lower pH values observed in the pork meat samples packaged with the commercial multilayer film can be explained by its lower oxygen permeance (2.15 × 10^−16^ m^3^·m^−2^·Pa^−1^·s^−1^ [21]) compared to the permeance values of the developed green composite films, which are in the range from 1.5 × 10^−15^ to 7.5 × 10^−16^ m^3^·m^−2^·Pa^−1^·s^−1^ [18], and this then could potentially delay the activity of spoilage microorganisms. The latter observation is also in accordance with the lower oxidation level and reduced total microbial counts of the pork meat packaged in these samples, as described below. Similar results were obtained in pork meat slices packaged in polylactide (PLA) films, showing pH values in the 5–6 range during the first week of cold storage [21].

The weight loss of the pork meat during storage is also reported in Figure 2b. The commercial multilayer lid film resulted in a significantly (*p* < 0.05) lower weight loss during storage compared to the pork meat samples packaged in trays with the green composite lid films. This weight variation can be ascribed to water evaporation of exudate that naturally occurs through the lid film, which is formed due to the leakage of intramuscular fluids from the cut surface [32]. Therefore, as seen in the figure note, this phenomenon was related to the different water vapor barrier properties of the evaluated packaging materials. Furthermore, one can observe that the weight loss was slightly lower, but still significant (*p* < 0.05), in the tray using the unfilled PHBV lid film due to the presence of celluloses which is known to increase water absorption and hence permeability of the biopolyester film [18]. Thus, after one week of storage, the meat sample packaged in the trays with compatibilized green composite resulted in the lowest weight loss (0.935%) among the PHBV-based lid films. This value was significantly lower (*p* < 0.05) than the weight loss values attained in the samples packaged using the neat PHBV film (1.117%), PHBV + TGIC + DCP film (1.488%), and PHBV/MFC film (1.489%). In any case, the weight losses attained were relatively low since these values were kept below 2%. For instance, pork meat fillets packaged in PLA films yielded values of weight loss of 3.4% after one week of storage, whereas these films were able to successfully preserve the food samples during this period under refrigeration conditions [21]. In another study, Konuk Takma and Kore [33] reported similar results for chicken breast meat packaged in PET films assembled with antimicrobial chitosan and alginate coatings, reaching values of 2–3% after five days of storage under cold storage conditions of 4 °C. In any case, one should also consider the effect of the packaging design and the fact that mass transfer is preferable in films to trays due to the higher area exposed to vapor and gas permeation.

Lipid oxidation in minced pork meat samples was evaluated by monitoring TBARS formation, which measures the amount of MDA produced by secondary products of polyunsaturated fatty acid peroxidation. The initial TBARS of the pork meat samples was 0.21 ± 0.09. As also shown in Figure 2c, the values obtained were consistent with the reported oxygen barrier properties of the polymer materials. Thus, the meat samples packaged in trays with the multilayer lid film showed the lowest oxidation level at the end of storage, that is, 0.38 MDA/kg, which was similar to that found in previous studies carried out with refrigerated pork meat slices [21]. It can also be observed that the TBARS values significantly (*p* < 0.05) decreased in the trays with the lids made of the MFC-containing PHBV films due to the higher oxygen barrier promoted by nanocelluloses. This result was particularly noticeable for the food sample packaged in the tray with the compatibilized green composite, having the lowest permeability among all the PHBV-based films tested herein. These samples yielded a value of 0.78 MDA/kg, which represents a reduction of approximately 30% when compared with the meat samples packaged with the lids of the neat PHBV films (1.08 MDA/kg) and PHBV + TGIC + DCP (1.18 MDA/kg). However, after one week of storage, all the pork meat samples packaged using PHBV films exceeded the threshold of 0.5 mg MDA/kg, which is habitually considered the limit of the detection of off-flavors in pork meat by consumers [34]. This result suggests that the shelf life of minced pork meat packaged in food trays with these lid films should not exceed 4–5 days when stored in refrigeration conditions.

**Figure 3:**
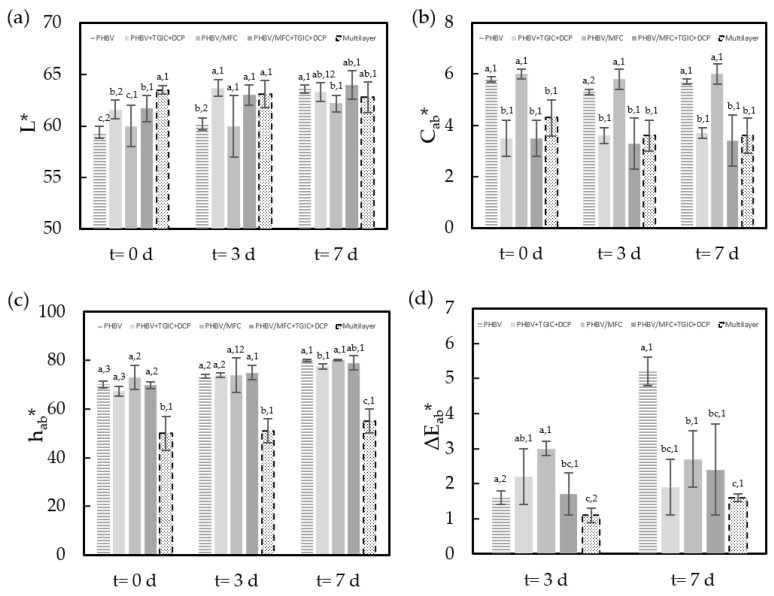
Development of color parameters in terms of (**a**) lightness (L*), (**b**) chroma (C_ab_*), (**c**) hue (h_ab_*), and (**d**) total color difference (ΔE_ab_*) of the cold-stored minced pork meat samples packaged in trays with the green composite lid films of poly(3-hydroxybutyrate-*co*-3-hydroxyvalerate) (PHBV) with microfibrillated cellulose (MFC) and compatibilized with triglycidyl isocyanurate (TGIC) and dicumyl peroxide (DCP) and the multilayer commercial film. Color parameters of each film reported in previous studies * [18] and ** [21]: L* (PHBV (88.41 ± 0.11)*; PHBV + TGIC + DCP (89.39 ± 0.15)*; PHBV/MFC (88.40 ± 0.30)*; PHBV/MFC + TGIC + DCP (88.86 ± 0.09)*; Multilayer (84.60 ± 1.00)**), C_ab_* (PHBV (10.24 ± 0.15)*; PHBV + TGIC + DCP (8.90 ± 0.40)*; PHBV/MFC (10.25 ± 0.99)*; PHBV/MFC + TGIC + DCP (9.40 ± 0.20)*; Multilayer (2.60 ± 0.40)**) and h_ab_* (PHBV (95.25 ± 0.11)*; PHBV + TGIC + DCP (98.00 ± 0.40)*; PHBV/MFC (95.10 ± 0.30)*; PHBV/MFC + TGIC + DCP (98.50 ± 0.20)*; Multilayer (132.00 ± 4.00)**). Different superscripts (a–c) indicate significant differences among samples for the same storage time and different superscripts (1–3) indicate differences due to storage time for the same sample (*p* < 0.05).

Furthermore, Figure 3 shows the evolution of the chromatic parameters (L*, C_ab_*, h_ab_*) and the total color difference (Δ*E*) of the minced pork meat with respect to the initial values (t = 0). Samples showed differences in chrome and hue at the beginning of storage as affected by the type of lid film used in the food tray. These differences can be mainly explained by the light scattering effect of each type of film on the meat sample according to the reported differences in their optical properties [18,21]. In fact, the average percentage of internal transmittance of the green composite samples ranged from 67% to 72% at 550 nm, whereas the commercial multilayer sample showed an average internal transmittance of 90% at this wavelength. One can further observe that the packaged meat samples L* and C_ab_* values hardly varied during the seven days of storage, whereas the h_ab_* values significantly (*p* < 0.05) increased. In this regard, one should consider that lipid oxidation and myoglobin oxidation are known to lead to discoloration in meat, and the two processes are frequently linked, since the oxidation of one of these compounds produces chemical species that promote the oxidation of the other [35]. In addition, the above-reported changes in the pH values can be associated with variations in the reduced form of myoglobin (Mb) and the susceptibility of muscle pigments to oxygenation and oxidation in pork meat. In particular, meat yellowness increases due to an increase in the relative amounts of myoglobin’s oxygenated and oxidized forms (MbO_2_ and MetMb) at the expense of the reduced form [36]. All these factors explain the reported significant (*p* < 0.05) color changes of the meat samples during the storage time. Furthermore, it is also worth mentioning that the samples packaged in the green composite films developed a redder hue and maintained the original color of the fresh meat. Finally, the total color differences after one week of storage of all the samples with respect to the initial day did not positively exceed the usual tolerance limit for food products (Δ*E* < 5 [37]), except for the samples packaged in the trays with the unfilled PHVB films, which rose to the 5-units level.

### 3.2. Microbial Characteristics of Packaged Minced Pork Meat

**Figure 4:**
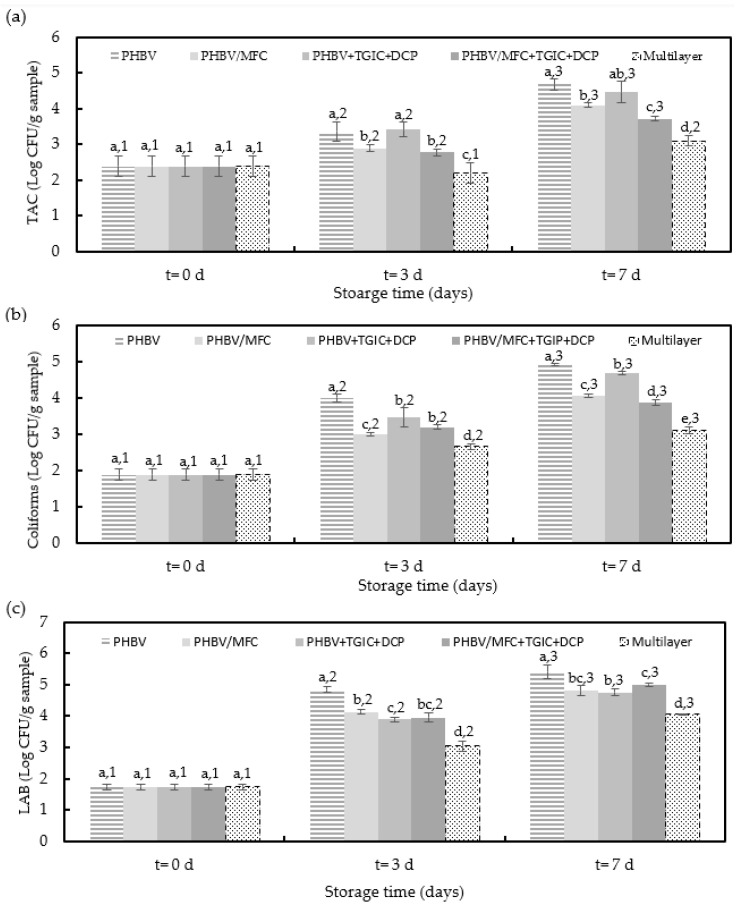
Total aerobic counts (TAC) (**a**), coliforms (**b**), and lactic acid bacteria (LAB) (**c**) of the cold-stored minced pork meat samples packaged in trays with the green composite lid films of poly(3-hydroxybutyrate-*co*-3-hydroxyvalerate) (PHBV) with microfibrillated cellulose (MFC) and compatibilized with triglycidyl isocyanurate (TGIC) and dicumyl peroxide (DCP) and the multilayer commercial film. Different superscripts (a–e) indicate significant differences among samples for the same storage time and different superscripts (1–3) indicate differences due to storage time for the same sample (*p* < 0.05).

Figure 4 shows the TAC, coliforms, and LAB counts of the packaged pork meat samples as a function of the storage time. As expected, the lowest microbial counts were observed in the meat samples packaged in trays with the commercial multilayer lid film due to its higher oxygen barrier. Similarly, the trays with the unfilled PHBV film yielded the meat samples with the highest bacterial growth, confirming the higher performance of the green composites. In many cases, differences among the meat samples packaged in the trays with PHBV-based lid films were only significant (*p* < 0.05) after three days of storage.

In terms of TAC, which is the quantitative standard for identifying the conditions and degree of contamination of meat [38], it can be observed that counts increased during refrigerated storage from approximately 2.4 logs CFU/g to values in the 3.7–4.7 CFU/g range for the minced pork meat packaged in the trays with the green composite lid films. Moreover, the bacterial growth pattern in the minced pork meat samples was similar to all the packaged meat samples, showing low growth during the first days. Differences among the meat samples were not significant (*p* > 0.05). Moreover, among the trays with PHBV-based films, the lowest bacterial counts were attained for the meat samples packaged with compatibilized green composites. Although the meat samples packaged in the trays with the multilayer lid films showed lower values throughout the storage period, none of the packaged minced pork samples exceeded the acceptable TAC level of five logs CFU/g. The latter value corresponds to the maximum acceptable level of TAC in mechanically separated fresh pork meat established by the European Commission (EC) in Regulation Number 2073/2005 [39]. In this regard, unpackaged pork meat fillets tested in the same conditions reached a TAC value above 7 logs CFU/g [21]. Therefore, according to these microbiological criteria, all evaluated packaging materials accomplished the requested value of food safety and quality to preserve pork meat.

Concerning the total coliforms, microbial counts increased from approximately 1.9 logs CFU/g to values ranging from 3.88 to 4.93 log CFU/g in the pork meat samples packaged in the trays with green composite lid films. It can be observed that the use of lid films of PHBV with MFC yielded minced pork samples with slightly lower coliform counts but still significant (*p* < 0.05) compared with the meat samples packaged in trays with PHBV and PHBV + TGIC + DCP films, whereas the multilayer lid yielded to the lowest bacterial counts. These results correlate well with the pH values reported above since total coliform counts are related to the permeance of oxygen gas through the lid film that favors the activity of spoilage microorganisms. These results were similar to those obtained in other studies with refrigerated packaged beef meat in PLA/PVA multilayer films [40].

Finally, microbial counts for LAB, which are the dominant group of microorganisms isolated from meat products [41], increased in the minced meat samples from 1.7 log CFU/g to values in the 4–5.5 log CFU/g range after one week of storage. As also observed for the TAC and coliform counts, the growth pattern was similar in all the pork meat samples, though different counts were attained. In the case of the meat samples packaged with the multilayer film, LAB counts were significantly (*p* < 0.05) lower than the values attained in the samples packaged using PHBV-based films. Similarly, the newly developed MFC-containing PHBV films successfully reduced the LAB counts of the meat samples, though differences were lower than 2 logs and thus showed no significant differences (*p* > 0.05). Similar results were obtained in pork meat slices packaged in PLA films, showing LAB values increased from 1.3 logs CFU/g to values in the 4–5 log CFU/g range after one week of storage [21].

### 3.3. Oxidative Stability of Packaged Sunflower Oil

**Figure 5:**
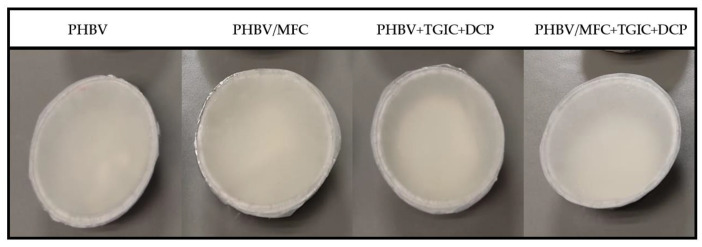
Images of the sunflower oil samples packaged in trays with the green composite lid films of poly(3-hydroxybutyrate-*co*-3-hydroxyvalerate) (PHBV) with microfibrillated cellulose (MFC) and compatibilized with triglycidyl isocyanurate (TGIC) and dicumyl peroxide (DCP).

The visual aspect of the sunflower oil packaged in the trays with the various PHBV-based lid films can be seen in Figure 5. All the samples showed a similar appearance due to the lack of transparency of the PHBV film.

**Figure 6:**
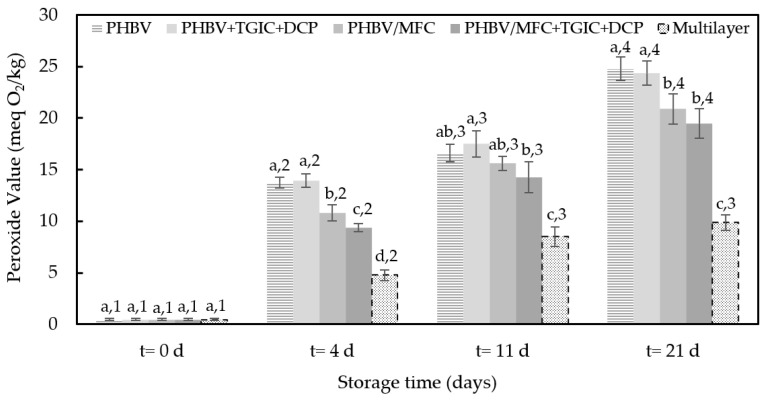
Peroxide values during accelerated storage conditions of the sunflower oil samples packaged in trays with the green composite lid films of poly(3-hydroxybutyrate-*co*-3-hydroxyvalerate) (PHBV) with microfibrillated cellulose (MFC) and compatibilized with triglycidyl isocyanurate (TGIC) and dicumyl peroxide (DCP) and the multilayer commercial film. Different superscripts (a–d) indicate significant differences among samples for the same storage time and different superscripts (1–4) indicate differences due to storage time for the same sample (*p* < 0.05).

Figure 6 shows the changes in PV of the sunflower oil samples during storage at 30 °C and 53% RH, with light to promote oil oxidation. The PV is associated with hydroperoxides derived from the primary oxidation of polyunsaturated fatty acids present in the sample. Thus, PV values are related to the initial oxidation stage [42]. It can be observed that the initial PV of sunflower oil was as low as 0.5 meq O_2_/kg, which is in the range of the detection limit, and then it showed a continuous increase during storage. Similar to that observed for pork meat, the oil samples packaged in trays with the multilayer lid films reached the lowest PV values due to their high-oxygen-barrier properties. In these food samples, the maximum PV of 10 meq O_2_/kg established in the Codex standard for fresh-named vegetable oils [43] was not exceeded throughout the storage period despite the accelerated oxidation conditions. In the case of the oil samples packaged with the PHBV-based lid films, after 21 days of storage, PV values reached levels between 19.5 and 24.8 meq O_2_/kg, showing the samples packaged with compatibilized green composite films had the lowest value.

Interestingly, and also in correlation to their higher oxygen barrier, the MFC-containing PHBV films induced significantly (*p* < 0.05) lower PV values in the packaged oil than the unfilled PHBV films, particularly in the case of the compatibilized green composite film. Moreover, these PV values are still below the 30–40 meq O_2_/kg range, which is generally associated with a rancid taste [44]. Moreover, an open aluminum cup containing 50 mL of sunflower oil was considered to reach values of 27 meq O_2_/kg, 74 meq O_2_/kg, and 130 meq O_2_/kg after 4, 11, and 21 days of storage, respectively. Similar results were obtained in other studies with the same oil and under the same accelerated conditions [26,45].

**Figure 7:**
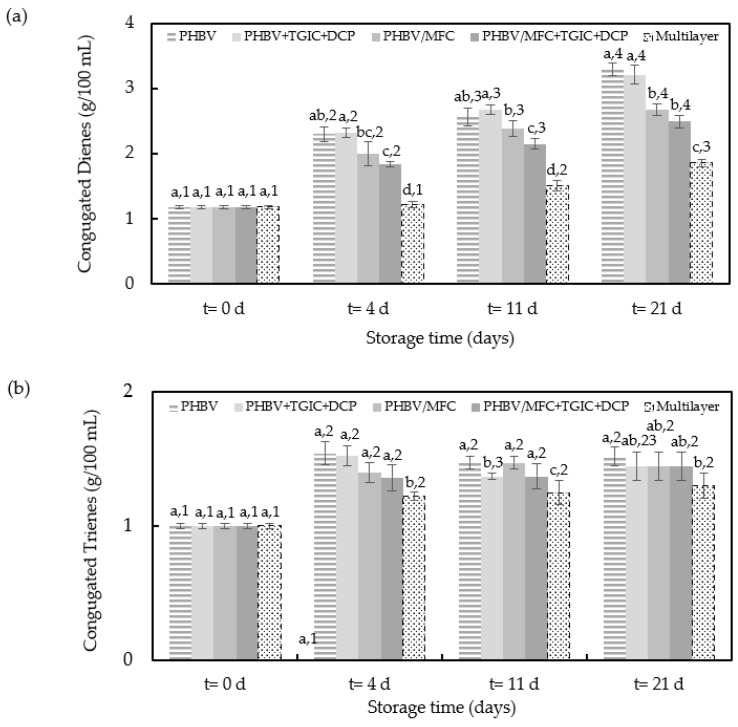
Conjugated dienes (**a**) and trienes (**b**) during accelerated storage conditions of the sunflower oil samples packaged in trays with the green composite lid films of poly(3-hydroxybutyrate-*co*-3-hydroxyvalerate) (PHBV) with microfibrillated cellulose (MFC) and compatibilized with triglycidyl isocyanurate (TGIC) and dicumyl peroxide (DCP) and the multilayer commercial film. Different superscripts (a–c) indicate significant differences among samples for the same storage time and different superscripts (1–4) indicate differences due to storage time for the same sample (*p* < 0.05).

The changes in the conjugated dienes and trienes content throughout the accelerated oxidation conditions are shown in Figure 7. Conjugated dienes and trienes are formed by the rearrangement of the hydroperoxide double bounds; thus, conjugated dienes represent the primary degradation products of oil that can be used to confirm the PV content, while conjugated trienes are related to the secondary products of the oxidation [46]. All samples showed increased conjugated dienes, with a pattern similar to those previously observed during PV analysis. However, the conjugated trienes remained almost constant during the storage period for the oil samples packaged in the trays with the multilayer and PHBV/MFC film lids, despite being significantly (*p* < 0.05) higher for the latter. This observation indicates that sunflower oil oxidation did not reach the second stage of degradation in these food trays. Therefore, these films prevented the occurrence of primary degradation products. The latter observation is in accordance with results reported in previous studies carried out under the same accelerated conditions [26]. Conversely, a significant (*p* < 0.05) increase was noticed for the samples packaged using unfilled PHBV films during the last days of the assays, confirming the higher oxidation level reached in these food trays. In particular, after three weeks of storage, samples packaged with the compatibilized green composite film presented the lowest conjugated dienes and trienes, showing values of 2.50 and 1.44 g/100 mL, respectively, whereas for the oil packaged with the neat PHBV film these were 3.30 and 1.52 g/100 mL. These differences can be mainly explained by the higher oxygen barrier achieved in the PHBV films by the presence of MFC, particularly when the dispersion was improved by the use of the TGIC and DCP compatibilizers.

### 3.4. Overall Migration in Food Simulants

The migration of the packaging constituents into the food is a prominent issue in food contact materials, especially for those containing nano and microfibers. Ethanol 10% *v*/*v* and olive oil, simulants A and D2, were chosen to simulate migration into minced pork meat and sunflower oil, respectively. The overall migration levels in the two evaluated foods, polar and non-polar simulants, are shown in Table 2.

All the evaluated PHBV-based films showed overall migration levels of 1–3 mg/dm^2^. Differences among the films were slight, though still significant (*p* < 0.05), which can be mainly related to sample heterogeneities but also to the potential release of MFC from the film surface. In any case, these values are well below 10 mg/dm^2^, which is the maximum level established for plastic materials and articles intended to come into contact with food for both simulants under the evaluated exposure conditions, complying with the overall migration limit (OML) set out in Commission Regulation (EU) No. 10/2011 on plastic materials and articles intended to come into contact with food and its subsequent amendments [28,29]. Other studies have also evaluated the overall migration level of PHBV reinforced with nanocelluloses in different food simulants. For instance, Li et al. [47] evaluated the migration of PHBV with cellulose nanocrystal-grafted rosin (CNC-R) in both 10% *v*/*v* ethanol and isooctane. Authors reported respective values of 161.5 and 127.1 µg/kg for each food simulant, both below the maximum limit of 60 mg/kg, whereas the presence of the nanocrystals reduced the migration due to their nucleation effect and resultant increment of crystallinity.

Similarly, Zhang et al. [48] also reported that the incorporation of cellulose nanocrystals-silver nanohybrids (CNC-Ag) into PHBV reduced the migration levels in 10% *v*/*v* ethanol and isooctane by approximately 50%, and all were well below the maximum migration limits (60 mg/kg of simulant). Furthermore, the conditions selected in the overall migration tests cover the contact of the packaging material with the foodstuff for all long-storage periods at room or lower temperatures. It also covers packaging filling at high-temperature conditions and/or packaging subjected to heating, from 70 °C for 2 h to 100 °C for 15 min. Therefore, it can be concluded that all the films tested can be safely applied to food applications to preserve foodstuff in polar and non-polar media. In any case, this will have to be further confirmed by specific migration studies according to the intended use and particular legislation.

## 4. Conclusions

The negative impact on the environment of commercial multilayer food packaging materials makes it necessary to find novel biodegradable alternatives based on biopolymers derived from renewable resources, such as the microbial copolymer PHBV, following the Circular Bioeconomy principles. In previous studies, novel green composites of PHBV reinforced with MFC and compatibilized with TGIC and DCP by a reactive melt-mixing process were developed and characterized in terms of their physical properties, yielding very promising materials for food preservation applications. In this study, the optimal formulations of the previously prepared green composites were applied as lid films in aluminum food trays to ascertain their capacity to preserve two fatty foods: minced pork meat and sunflower oil. Results showed that the PHBV-based reinforced green composites effectively preserved the physicochemical and microbiological quality of minced pork meat samples during storage for one week at 5 °C. Regarding the prevention of oxidation of sunflower oil, all evaluated green composites also showed satisfactory performance in avoiding the formation of peroxide levels associated with a rancid taste and in preventing the occurrence of primary degradation products in accelerated storage conditions. For both fatty foods, the compatibilized green composite films accomplished higher values of food quality than the unfilled PHBV films, which was ascribed to the higher oxygen barrier attained in the copolyester by incorporating atomized nanocelluloses. Moreover, all PHBV-based films proved to be safe for their packaging application to preserve meat and oil products since their overall migration levels did not exceed in either of the two simulants (10% *v*/*v* ethanol and vegetable oil) the maximum level established by legislation. However, specific migration studies should be further conducted to confirm their safe use in food preservation applications according to particular legislation.

## Figures and Tables

**Table 1:** Summary of film compositions according to the weight content (wt%) of poly(3-hydroxybutyrate-*co*-3-hydroxyvalerate) (PHBV) and microfibrillated cellulose (MFC) in which triglycidyl isocyanurate (TGIC) and dicumyl peroxide (DCP) were added as parts per hundred resins (phr) of green composite.

Sample	PHBV (wt%)	MFC (wt%)	TGIC (phr)	DCP (phr)
PHBV	100	0	0	0
PHBV/MFC	95	5	0	0
PHBV + TGIC + DCP	100	0	1	0.25
PHBV/MFC + TGIC + DCP	95	5	1	0.25

**Table 2:** Overall migration levels of the green composite films of poly(3-hydroxybutyrate-*co*-3-hydroxyvalerate) (PHBV) with microfibrillated cellulose (MFC) and compatibilized with triglycidyl isocyanurate (TGIC) and dicumyl peroxide (DCP) into food simulants.

Film	Ethanol 10% *v*/*v* (mg/dm^2^)	Vegetable Oil (mg/dm^2^)
PHBV	1.7 ± 0.2 ^a^	2.2 ± 0.1 ^a^
PHBV/MFC	1.9 ± 0.5 ^a^	2.6 ± 0.2 ^b^
PHBV/MFC + TGIC + DCP	2.8 ± 0.4 ^b^	2.7 ± 0.3 ^b^

Different superscripts within the same column indicate significant differences among samples (^a,b^) (*p* < 0.05).

## Data Availability

Data is contained within the article and available on request.
